# Psychometric network analysis of depression in hypertensive older adults: identifying core symptoms and modifiable risk factors

**DOI:** 10.3389/fpsyt.2026.1751228

**Published:** 2026-02-04

**Authors:** Yue Li, Fengdan Chen, Lanxian Mai, Haibin Luo, Zhiyu Zeng

**Affiliations:** 1 Department of Cardiology, The First Affiliated Hospital of Guangxi Medical University, Nanning, Guangxi, China; 2 Guangxi Key Laboratory Base of Precision Medicine in Cardio-Cerebrovascular Diseases Control and Prevention, Guangxi Clinical Research Center, Nanning, Guangxi, China; 3 Department of Geriatric Endocrinology, The First Affiliated Hospital of Guangxi Medical University, Nanning, Guangxi, China

**Keywords:** central symptoms, depression, hypertension, network analysis, older adults, risk factors

## Abstract

**Objectives:**

This study aims to construct a depression symptom network in elderly hypertensive patients, identify central and bridging symptoms, and explore the association between network structure and modifiable risk factors.

**Methods:**

This study adopts a retrospective research design, reviewing the medical records and survey data of 562 elderly hypertensive patients from a tertiary comprehensive hospital from September 2022 to May 2023. The data was retrospectively collected from patient health records including a general demographic questionnaire, Insomnia Severity Index-7(ISI-7), 9-item Patient Health Questionnaire (PHQ-9), Generalized Anxiety Disorder-7 (GAD-7), and Connor Davidson Resilience Scale-25 (CD-RISC-25). Calculate centrality indices (intensity, betweenness centrality, and intimacy) to identify core symptoms. A comprehensive network model integrating GAD-7, ISI-7, CD-RISC-25, and demographic variables was constructed.

**Results:**

A total of 562 patients were enrolled in the study. The average score of PHQ-9 is (10.69 ± 3.42) points. Network analysis shows that anhedonia (PHQ1) exhibits the highest intensity centrality. The strongest partial correlation was observed between Sleep problems(PHQ3) and PHQ1 (weight=0.40), fatigue (PHQ4) and depressed mood (PHQ2) (weight=0.29), and PHQ4 and PHQ1 (weight=0.29). There are two different symptom clusters: somatic affective clusters (PHQ1, PHQ3, PHQ4) and cognitive vegetative clusters (appetite problems(PHQ5), feeling of worthlessness (PHQ6), concentration problems (PHQ7)). Suicide ideation (PHQ9) exhibits the lowest centrality. The comprehensive network model indicates a strong positive correlation between depression and anxiety (PHQ-GAD), depression and insomnia (PHQ-ISI), and anxiety and insomnia (GAD-ISI). The dimensions of psychological resilience, including self reinforcement, resilience, and optimism, are negatively correlated with PHQ scores (all P<0.001), while GAD-7 scores are positively correlated. There are edge connections between exercise (EX) and ISI, disease course (DU), and gender (GD). Drink (DR) is positively correlated with GD, while degree of education (DOE) is connected within demographic clusters and has an edge with GD.

**Conclusions:**

Network analysis revealed that in the depressive network of patients with hypertension, anhedonia is the most central symptom, indicating that it may become a primary intervention target. The comprehensive network uncovered significant interconnections among depression, anxiety, and insomnia. Furthermore, the resilience dimension negatively correlates with depressive symptoms, while there are edge connections between exercise and both insomnia and demographic factors, highlighting modifiable protective factors.

## 1 Introduction

Hypertension affects approximately 1.28 billion adults worldwide, with the highest prevalence observed in individuals aged 60 years and older (1). Research suggests that a history of hypertension can affect the 24-hour mean blood pressure and blood pressure variability in patients with acute ischemic stroke (AIS) undergoing endovascular thromboectomy (EVT), thereby influencing the prognosis of their functional outcomes (2). Beyond its well-established cardiovascular consequences, hypertension has gained increasing recognition as a significant risk factor for mental health disorders, particularly depression (3). Studies indicate that older adults with hypertension exhibit clinically significant depressive symptoms at rates considerably higher than their normotensive counterparts (3, 4). While the association between hypertension and depression in older populations is acknowledged, conventional research approaches have largely conceptualized depression as a unidimensional construct, relying on total scores from standardized instruments for assessment (5). This approach, though useful for screening purposes, obscures the inherent heterogeneity of depressive presentations and fails to elucidate the intricate interrelationships among individual symptoms (6). Depression encompasses a diverse array of manifestations, spanning cognitive-affective symptoms such as depressed mood and anhedonia to somatic complaints including fatigue and sleep disturbances. These symptoms may exhibit distinct patterns of interaction in older adults living with chronic physical conditions (6, 7). Elucidating the dynamic architecture of these symptom relationships is essential for informing targeted intervention strategies; however, research specifically addressing this question in older hypertensive populations remains limited.

Network analysis represents a paradigm shift in psychopathology research, offering a framework that conceptualizes mental disorders as dynamic systems of causally interacting symptoms rather than manifestations of discrete latent entities (8). Within this approach, individual symptoms are modeled as nodes, and their pairwise associations as edges, allowing researchers to identify core symptoms that exert disproportionate influence on the overall network structure (9).These central symptoms are theorized to play a pivotal role in both the onset and persistence of depressive episodes, rendering them prime candidates for targeted intervention (10). Notably, the architecture of depressive symptom networks may vary substantially across populations, shaped by differences in underlying vulnerabilities, comorbidity profiles, and sociodemographic characteristics. To date, network-based investigations of depression in older hypertensive patients remain scarce, despite the unique physiological susceptibilities and psychosocial stressors characteristic of this population (11). Existing research in this domain has primarily focused on network models examining insomnia, anxiety, and depression among community residents and healthcare workers during the COVID-19 pandemic (12, 13).

Identifying core symptoms and elucidating how lifestyle and psychosocial factors shape symptom network configurations hold particular relevance for developing feasible, cost-effective interventions in clinical practice (10). Establishing evidence-based priorities for such interventions, however, necessitates a granular understanding of symptom-level mechanisms and the contextual factors that modulate them (14). The present retrospective study seeks to address these gaps through a comprehensive network analysis of depressive symptoms in older adults with hypertension, drawing on previously collected clinical data. Specifically, we aimed to: (1) construct and characterize a depressive symptom network based on Patient Health Questionnaire-9 (PHQ-9) assessments documented in existing records; (2) identify central symptoms as well as bridge symptoms connecting distinct symptom clusters; (3) employ network comparison tests to examine associations between modifiable risk factors and network structural properties; and (4) explore potential mechanisms through which these risk factors may influence specific symptom interconnections.

## 2 Participants and methods

### 2.1 Participants

This study adopts a retrospective research design. A retrospective review and analysis were conducted on the medical records and previously collected survey data of 974 hypertensive patients in a tertiary comprehensive hospital from September 2022 to May 2023.

Inclusion criteria: According to medical record review, patients who meet the following criteria are eligible for inclusion: (1) age ≥ 60 years old at the time of data collection; (2) According to the medical records, the clinical diagnosis is primary hypertension (defined as systolic blood pressure ≥ 140 mmHg and/or diastolic blood pressure ≥ 90 mmHg, or with a record of antihypertensive drug use) for at least 6 months; (3) Written proof of Mandarin communication skills; (4) Provide informed consent documents during the collection of raw data.

Exclusion criteria include: (1) diagnosis of secondary hypertension recorded in medical records; (2) Serious cognitive impairment recorded in clinical documents; (3) Diagnosis of mental disorders, bipolar disorder, or other serious mental illnesses recorded in medical records; (4) Acute cardiovascular events (such as myocardial infarction, stroke) that occurred within the 3 months prior to the evaluation date; (5) The attending physician previously assessed a terminal illness or life expectancy of less than 6 months; (6) Records of participation in other clinical trials during the research period; (7) The questionnaire data is incomplete (missing items in retrospective evaluation>20%).

### 2.2 Sample size determination

Sample size determination was based on recommendations for network analysis, which suggest a minimum of 3 participants per estimated paramete (8). Given the PHQ-9’s 9 items yielding 36 unique edges in a fully connected network, a minimum of 500 cases was required. Through systematic review of medical records, we identified 974 potentially eligible patients who had completed relevant assessments during the study period. After applying inclusion and exclusion criteria, 412 patients were excluded due to incomplete questionnaires or failure to meet eligibility requirements, resulting in a final sample of 562 patients for analysis.This retrospective study protocol was approved by the Institutional Review Board of our center (2025-E1049). All data extracted for this retrospective analysis were anonymized using unique identification codes, and all records were stored in password-protected databases accessible only to authorized research team members. Researchers underwent standardized training prior to data collection, established a dual-core data entry system, and employed logical validation and range validation to identify outliers.

### 2.3 Measures

#### 2.3.1 Demographic basic information

Collect sociodemographic and clinical variables through medical record review. Sociodemographic factors such as age, gender, education level, marital status, and living arrangements. Clinical factors: duration of hypertension (years since diagnosis), systolic and diastolic blood pressure (average of three measurements taken at 2-minute intervals using a calibrated automatic blood pressure monitor [Omron HEM-7200].

#### 2.3.2 Patient health questionnaire-9

The PHQ-9 was used to assess depressive symptoms (15). This 9-item self-report instrument evaluates the presence and severity of each DSM-5 major depressive disorder criterion over the past 2 weeks. Items include: (1) anhedonia, (2) depressed mood, (3) sleep problems, (4) fatigue, (5) appetite problems, (6) Feeling of worthlessness, (7) concentration problems, (8) psychomotor changes, and (9) suicidal ideation. Each item is rated on a 4-point Likert scale (0=“not at all” to 3=“nearly every day”), yielding total scores ranging from 0 to 27. The Chinese version of PHQ-9 has demonstrated excellent psychometric properties in older adult populations (16).

#### 2.3.3 Insomnia severity index-7

The ISI-7 has become one of the widely used sleep assessment tools in current clinical practice (17). The scale consists of 7 items, covering difficulty falling asleep, difficulty maintaining sleep, early awakening problems, satisfaction with sleep, concerns about sleep problems, the degree of impact on daily functioning, and the impact on quality of life. A higher score indicates a greater severity of insomnia. If the total ISI score exceeds 7, it is considered clinically significant insomnia symptoms. The ISI scale has good internal consistency and reliability tests, and is recommended as a clinical tool for assessing insomnia patients in China (18).

#### 2.3.4 Generalized anxiety disorder-7

The GAD-7 is a scale used to assess the degree and frequency of negative emotions experienced by participants over the past two weeks (19). The scale consists of 7 items, with a total score ranging from 0 to 21 points, where higher scores indicate greater severity.The GAD-7 scale has good internal consistency and reliability tests, and is recommended as a clinical tool for patients in China (16).

#### 2.3.5 Psychological resilience scale-25

The CD-RISC-25 is a psychological resilience assessment tool developed by Connor and Davidson (20). The Chinese version of CD-RISC comprises three dimensions with 25 items. These dimensions include: Optimism, reflecting an individual’s hopeful and confident outlook on future development; Self-enhancement, representing a positive and courageous attitude toward overcoming setbacks; and Resilience, indicating the ability to maintain mental toughness and withstand stress in adversity. Each item on CD-RISC is scored on a scale from “never” to “always.” The scale’s scores positively correlate with psychological resilience levels, meaning higher scores indicate stronger resilience. The Chinese version of CD-RISC demonstrates excellent reliability and validity, having undergone rigorous validation.

### 2.4 Data collection procedures

Data for this retrospective study were extracted from previously collected assessments administered between September 2022 and May 2023. The original data collection had been conducted by trained research nurses who had completed a standardized 8-hour training program covering ethical principles, informed consent procedures, questionnaire administration, and data quality assurance. Quality control measures implemented during the original data collection included: (1) immediate review of completed questionnaires for missing items; (2) weekly data entry verification by two independent researchers with discrepancies resolved through consensus; (3) 10% random double-entry validation (consistency rate: 99.3%); and (4) monthly supervision meetings to address procedural issues. For this retrospective analysis, data extraction was performed by two independent researchers, with 100% concordance achieved through consensus discussion. Data integrity was verified by cross-checking extracted data against original paper records and electronic databases.

### 2.5 Statistical analysis

All analyses were conducted using SPSS26.0 and R version 4.3.1. Statistical significance was set at α=0.05 (two-tailed) unless otherwise specified.

#### 2.5.1 Descriptive statistics

Continuous variables were summarized as means and standard deviations (SD) or medians and interquartile ranges (IQR) depending on normality assessed by Shapiro-Wilk tests and visual inspection of Q-Q plots. Categorical variables were presented as frequencies and percentages. Independent samples t-tests, Mann-Whitney U tests, chi-square tests, or Fisher’s exact tests were used to compare characteristics between groups with and without clinically significant depressive symptoms.

#### 2.5.2 Network estimation

Network models were estimated using the bootnet package (version 1.5) and visualized using the qgraph package (version 1.9.5) (21). Implemented the following program: regularized partial correlation network. Use graphical LASSO and extended Bayesian Information Criterion (EBIC) model selection to estimate Gaussian Graph Model (GGM). This method estimates the regularized partial correlation between all PHQ-9 item pairs and controls all other items in the network. The regularization parameter (λ) is selected through EBIC, and the hyperparameter γ=0.5 balances the sparsity and goodness of fit of the model. The edge represents the unique paired associations between symptoms after excluding all other symptoms, and the edge weight represents the strength of conditional dependence. Three centrality indices were calculated to identify influential symptoms: intensity centrality: the sum of absolute edge weights connected to nodes, indicating the degree of direct correlation between symptoms and other symptoms; Tight centrality: The reciprocal of the sum of the shortest path lengths from one node to all other nodes, reflecting the direct connection between symptoms and other symptoms in the network; Intermediate centrality: The number of times a node is located on the shortest path between two other nodes, used to identify bridge symptoms that connect symptom clusters. Network stability and accuracy: Non parametric bootstrap (5000 iterations) is used to evaluate network stability and estimate the 95% confidence interval (CI) around edge weights. Evaluate the accuracy of edge weights by calculating the proportion of bootstrap samples with significantly different edge weights from zero. Use the case deletion subset bootstrap program (1000 iterations) to check centrality stability(CS coefficient ≥ 0.50 is the acceptable threshold, ≥ 0.70 is the ideal threshold (9)). The program sequentially removes the percentage increase of cases (10% -75%) and recalculates the centrality index. Use bootstrap confidence intervals to perform edge weight difference tests and centrality difference tests to determine which edge and centrality values are significantly different from each other.

## 3 Results

### 3.1 Univariate analysis of demographic data and PHQ symptom scores in hypertensive patients

The final sample comprised 562 hypertensive older adults. Participants’ blood pressure grading at admission showed 31.0% Grade 1 hypertension, 15.5% Grade 2, and 5.5% Grade 3. Statistically significant differences were observed in patients’ gender, disease duration, admission blood pressure, degree of education, drink, exercise and PHQ scores (P<0.05). Detailed research results are shown in **Table 1**.

**Table 1:** Univariate analysis of demographic data and PHQ-9 symptom scores in hypertensive patients.

Variable	Category	Frequency (%)	PHQ-9 (Mean ± SD)	*t/F*	*P*
gender	male	370(65.8)	10.37 ± 3.51	-3.067	0.002
	female	192(34.2)	11.30 ± 3.17		
nation	the Han nationality	386(68.7)	10.80 ± 3.34	1.507	0.222
	the Zhuang nationality	157(27.9)	10.56 ± 3.62		
	other	19(3.4)	9.47 ± 3.29		
habitation	countryside	145(25.8)	10.57 ± 3.16	0.710	0.492
	town	144(25.6)	10.98 ± 3.71		
	city	273(48.6)	10.60 ± 3.40		
degree of education	Primary school and below	256(45.6)	9.84 ± 3.44	10.070	<0.001
	junior middle school	176(31.3)	11.41 ± 2.83		
	senior middle school	82(14.6)	11.38 ± 3.67		
	College degree or above	48(8.5)	11.38 ± 3.89		
marital	unmarried	2(0.4)	13.00 ± 5.66	0.368	0.776
	married	541(96.3)	10.69 ± 3.39		
	dissociaton	4(0.7)	11.00 ± 3.65		
	bereft of one’s spouse	15(2.7)	10.33 ± 4.41		
Current work status	be on the job	164(29.2)	10.65 ± 3.24	1.513	0.221
	Leave/Retire	236(42.0)	10.95 ± 3.72		
	other	162(28.8)	10.35 ± 3.11		
medical insurance	Residents’ Medical Insurance	400(71.2)	10.73 ± 3.52	0.285	0.752
	Employee Medical Insurance	113(20.1)	10.48 ± 3.25		
	at one’s own expense	49(8.7)	10.84 ± 2.96		
smoke	No	379(67.4)	10.49 ± 3.45	1.953	0.143
	Yes	135(24)	11.13 ± 3.13		
	quit smoking	48(8.5)	10.98 ± 3.90		
drink	No	390(69.4)	10.36 ± 3.52	6.291	0.002
	Yes	106(18.9)	11.26 ± 3.08		
	abstinence	66(11.7)	11.70 ± 3.02		
Drinking strong tea and coffee	No	274(48.8)	10.49 ± 3.43	-1.362	0.174
	Yes	288(51.2)	10.88 ± 3.40		
Exercise (e.g., jogging, walking, etc.)	0 times per week	243(43.2)	11.30 ± 3.46	16.715	<0.001
	1–2 times per week	108(19.2)	11.36 ± 3.22		
	≥3 times per week	211(37.5)	9.64 ± 3.21		
Disease duration (years)	<5 years	221(39.3)	9.57 ± 3.49	1.402	0.241
	5 years ≤ disease duration ≤ 10 years	246(43.8)	11.36 ± 2.95	13.895	<0.001
	10 years <disease course ≤ 15 years	25(4.4)	11.88 ± 3.21		
	Over 15 years	70(12.5)	11.40 ± 3.87		
Blood pressure grading at admission	normal	270(48.0)	10.19 ± 3.31	4.302	0.005
	I level	174(31.0)	11.08 ± 3.515		
	II level	87(15.5)	11.47 ± 3.33		
	III level	31(5.5)	10.65 ± 3.57		

### 3.2 Correlation analysis between psychological factors and PHQ-9 symptom scores in hypertensive patients

In terms of psychological factors, the PHQ-9 symptom score (10.69 ± 3.42) showed a negative correlation with the dimensions of psychological resilience such as self strengthening (25.35 ± 3.19), resilience (36.41 ± 5.36), and optimism (13.05 ± 1.87), and a positive correlation with the GAD-7 score (4.23 ± 2.61), all of which were statistically significant (P<0.001). The remaining detailed results are shown in **Table 2**.

**Table 2:** Correlation analysis between psychological factors and PHQ-9 symptom scores in hypertensive patients.

Variable		ISI-7	GAD-7	Self-improvement	Tenacious	Optimism	CD-RISC-25	PHQ-9
ISI-7	r	1						
GAD-7	r	0.725^**^	1					
Self-improvement	r	-0.490^**^	-0.503^**^	1				
Tenacious	r	-0.471^**^	-0.515^**^	0.765^**^	1			
Optimism	r	-0.408^**^	-0.454^**^	0.592^**^	0.512^**^	1		
CD-RISC-25	r	-0.523^**^	-0.562^**^	0.906^**^	0.945^**^	0.702^**^	1	
PHQ-9	r	0.454^**^	0.528^**^	-0.384^**^	-0.428^**^	-0.310^**^	-0.443^**^	1
	P	<0.001	<0.001	<0.001	<0.001	<0.001	<0.001	

** The correlation is significant at the 0.01 level (two-tailed). Patient Health Questionnaire-9 (PHQ-9), Generalized Anxiety Disorder Scale-7 (GAD-7), nsomnia Severity Index-7 (ISI-7), Psychological Resilience Scale (CD-RISC-25).

### 3.3 PHQ Symptom network model for hypertension patients

#### 3.3.1 Gaussian partial correlation network model

**Figure 1:**
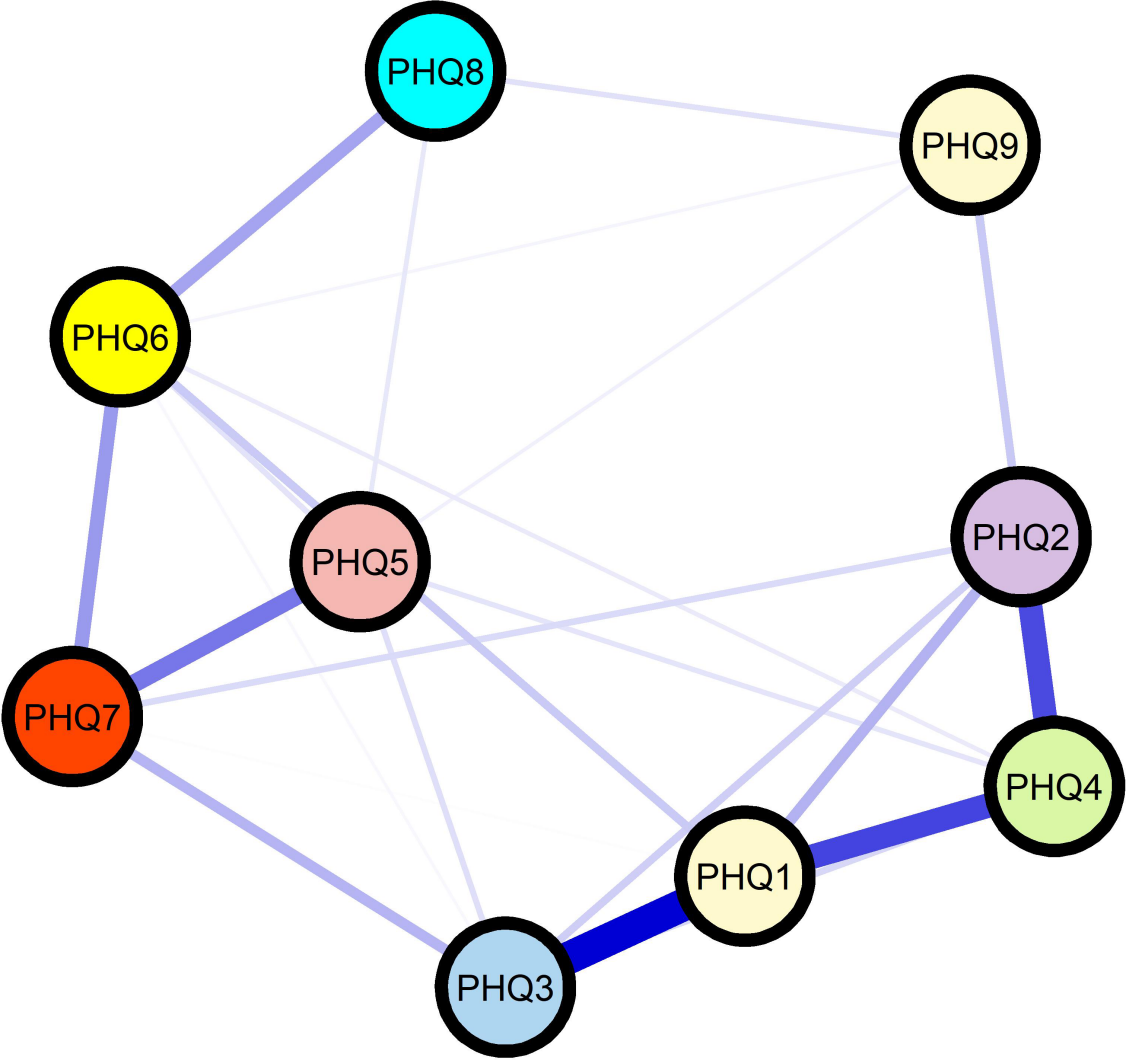
Network structure of depressive symptoms in hypertensive older Adults. Network visualization of Patient Health Questionnaire-9 (PHQ-9) items in hypertensive older adults (N = 562). Nodes represent individual depressive symptoms: PHQ1 (Anhedonia), PHQ2 (Depressed mood), PHQ3 (Sleep problems), PHQ4 (Fatigue), PHQ5 (Appetite problems), PHQ6 (Feeling of worthlessness), PHQ7 (Concentration problems), PHQ8 (Psychomotor changes), and PHQ9 (Suicidal ideation). Node colors differentiate symptom clusters based on community detection algorithms. Edge thickness represents the strength of regularized partial correlations between symptoms, with thicker edges indicating stronger conditional associations. Edge colors indicate the direction of associations: blue edges represent positive associations, and red edges represent negative associations (if present).

The network consisted of 9 nodes and 21 edges. The strongest edges were observed between PHQ3 (sleep problems) and PHQ1 (anhedonia) (weight = 0.40), PHQ4 (fatigue) and PHQ2 (depressed mood) (weight = 0.29), and PHQ4 and PHQ1 (weight =0.29). The estimated network structure of the PHQ-9 symptoms is presented in **Figure 1**.

#### 3.3.2 Network node centrality index

**Figure 2:**
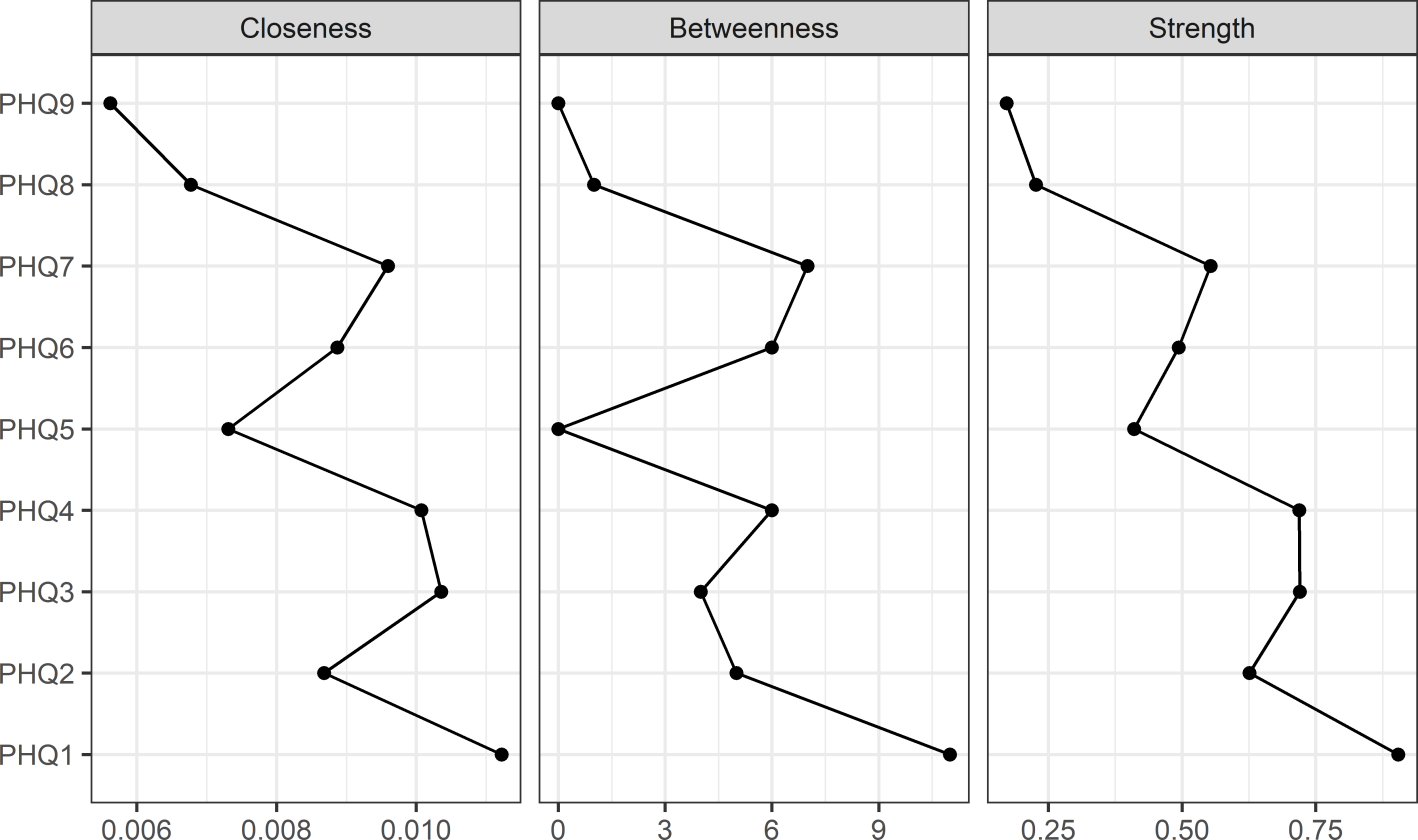
Centrality indices of depressive symptoms network. Standardized centrality measures for PHQ-9 symptoms in the depression network. Closeness centrality indicates how directly a symptom connects to other symptoms in the network, with higher values suggesting greater overall connectivity; Betweenness centrality reflects the extent to which a symptom lies on the shortest path between other symptoms, with higher values indicating greater bridging function in symptom activation pathways; Strength centrality represents the sum of absolute edge weights connected to each symptom, with higher values indicating stronger overall connectivity with other symptoms. PHQ-9 (Patient Health Questionnaire-9), PHQ1 (Anhedonia), PHQ2 (Depressed mood), PHQ3 (Sleep problems), PHQ4 (Fatigue), PHQ5 (Appetite problems), PHQ6 (Feeling of worthlessness), PHQ7 (Concentration problems), PHQ8 (Psychomotor changes), and PHQ9 (Suicidal ideation).

Three standardized centrality measures were computed to identify the most central symptoms in the network in **Figure 2**. For strength centrality, which reflects the overall connectivity of each symptom, PHQ1 demonstrated the highest value, followed by PHQ7 (concentration problems) and PHQ4. In contrast, PHQ9 (suicidal ideation) exhibited the lowest strength centrality, indicating its relatively peripheral position in the network.

#### 3.3.3 Edge weight matrix and differential analysis

**Figure 3:**
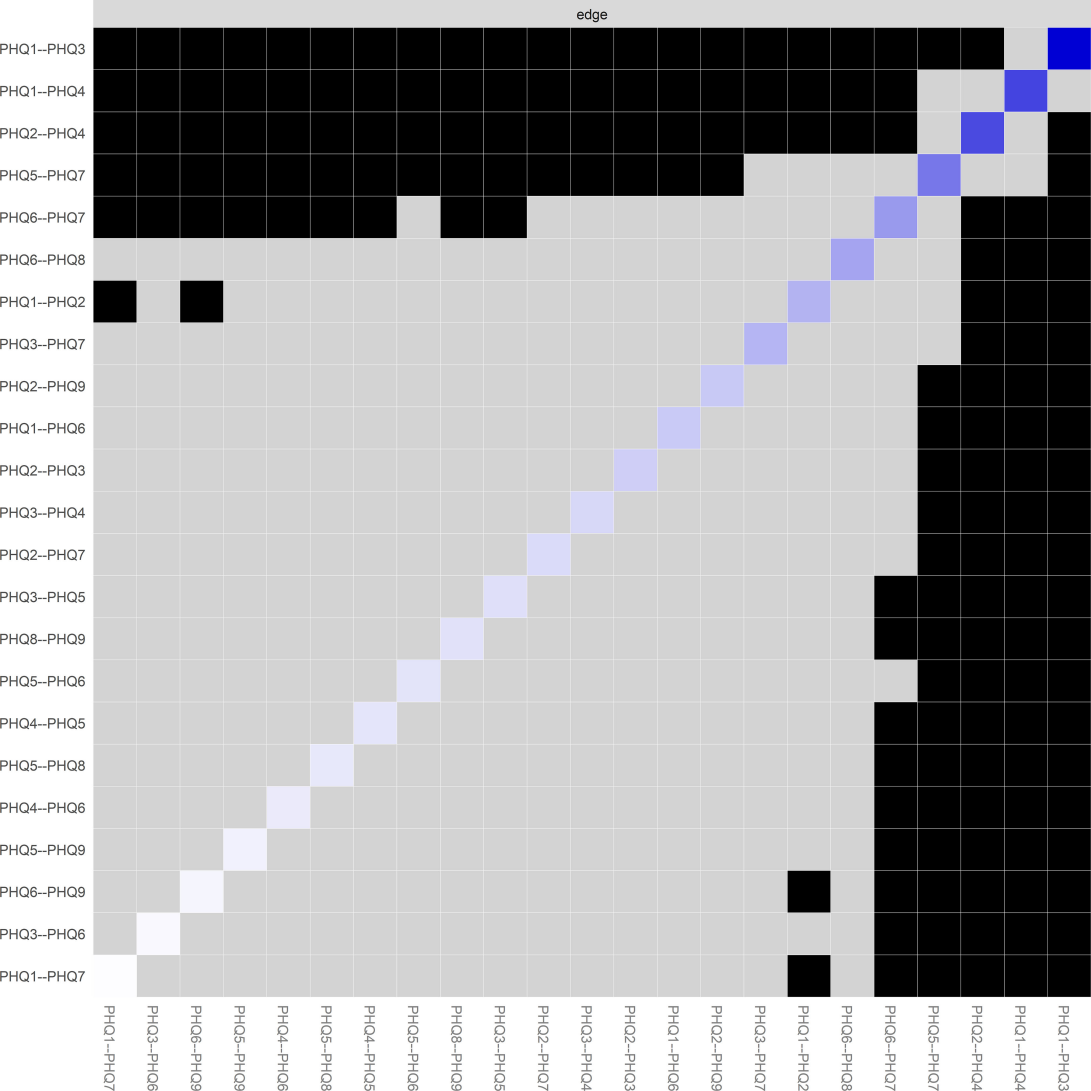
PHQ-9 symptoms edge weight matrix and differential analysis. Heatmap visualization of edge weights in the depression symptom network. The matrix displays the strength of connections between all pairs of PHQ-9 symptoms. The color gradient ranges from white (weak or absent connections) to dark blue (strong positive connections), with black indicating the strongest associations. Each cell represents the regularized partial correlation between two symptoms after controlling for all other symptoms in the network. PHQ-9 (Patient Health Questionnaire-9), PHQ1 (Anhedonia), PHQ2 (Depressed mood), PHQ3 (Sleep problems), PHQ4 (Fatigue), PHQ5 (Appetite problems), PHQ6 (Feeling of worthlessness), PHQ7 (Concentration problems), PHQ8 (Psychomotor changes), and PHQ9 (Suicidal ideation).

The complete matrix of edge weights is displayed in **Figure 3**. The Strong positive connections were predominantly observed among PHQ1, PHQ3, and PHQ4, forming a somatic-affective symptom cluster. Another notable cluster emerged around PHQ5 (appetite problems), PHQ6 (worthlessness), and PHQ7, representing a cognitive-vegetative dimension. Bootstrap confidence intervals (95% CI) for the strongest edges indicated robust estimation: PHQ1-PHQ3, PHQ2-PHQ4, and PHQ1-PHQ4.

#### 3.3.4 Stability of centrality indices assessed by case-dropping subset bootstrap analysis

**Figure 4:**
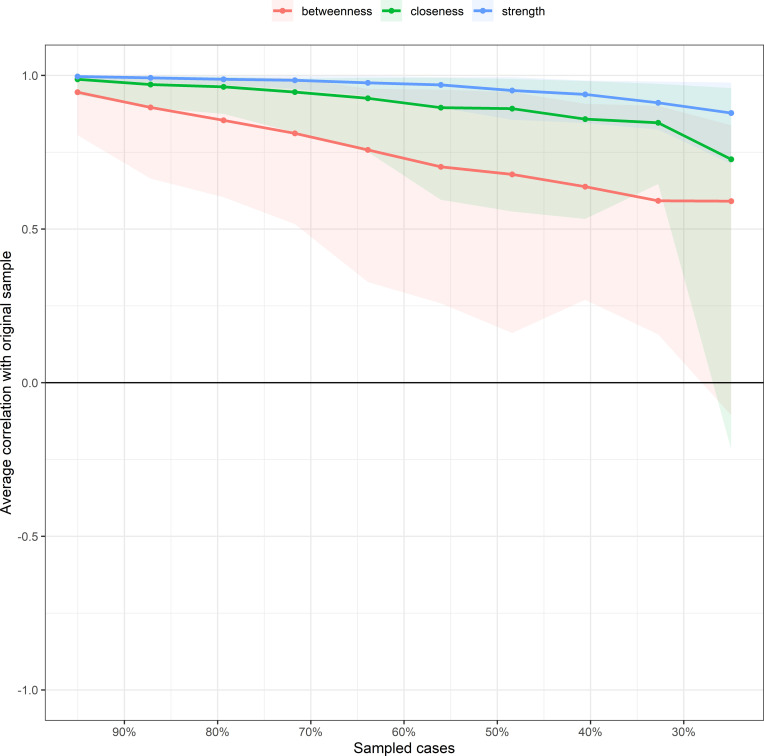
Stability of centrality indices assessed by case-dropping subset bootstrap analysis. This figure displays the average correlation between centrality indices obtained from the original sample and those from subsets with progressively smaller proportions of cases. The x-axis represents the percentage of sampled cases (ranging from 95% to 25%), while the y-axis represents the average correlation with the original sample. Three centrality indices are presented: strength (blue line), closeness (green line), and betweenness (red line).

The stability of centrality indices was evaluated using the case-dropping subset bootstrap procedure with 1, 000 iterations. As illustrated in **Figure 4**, the average correlations between centrality indices derived from the original sample and those from subsets with progressively reduced sample sizes were examined. Results suggest that strength centrality demonstrated the highest stability (CS-coefficient > 0.7), followed by closeness, while betweenness centrality showed relatively lower stability.

#### 3.3.5 Integrated network model with anxiety, insomnia, and modifiable risk factors

**Figure 5:**
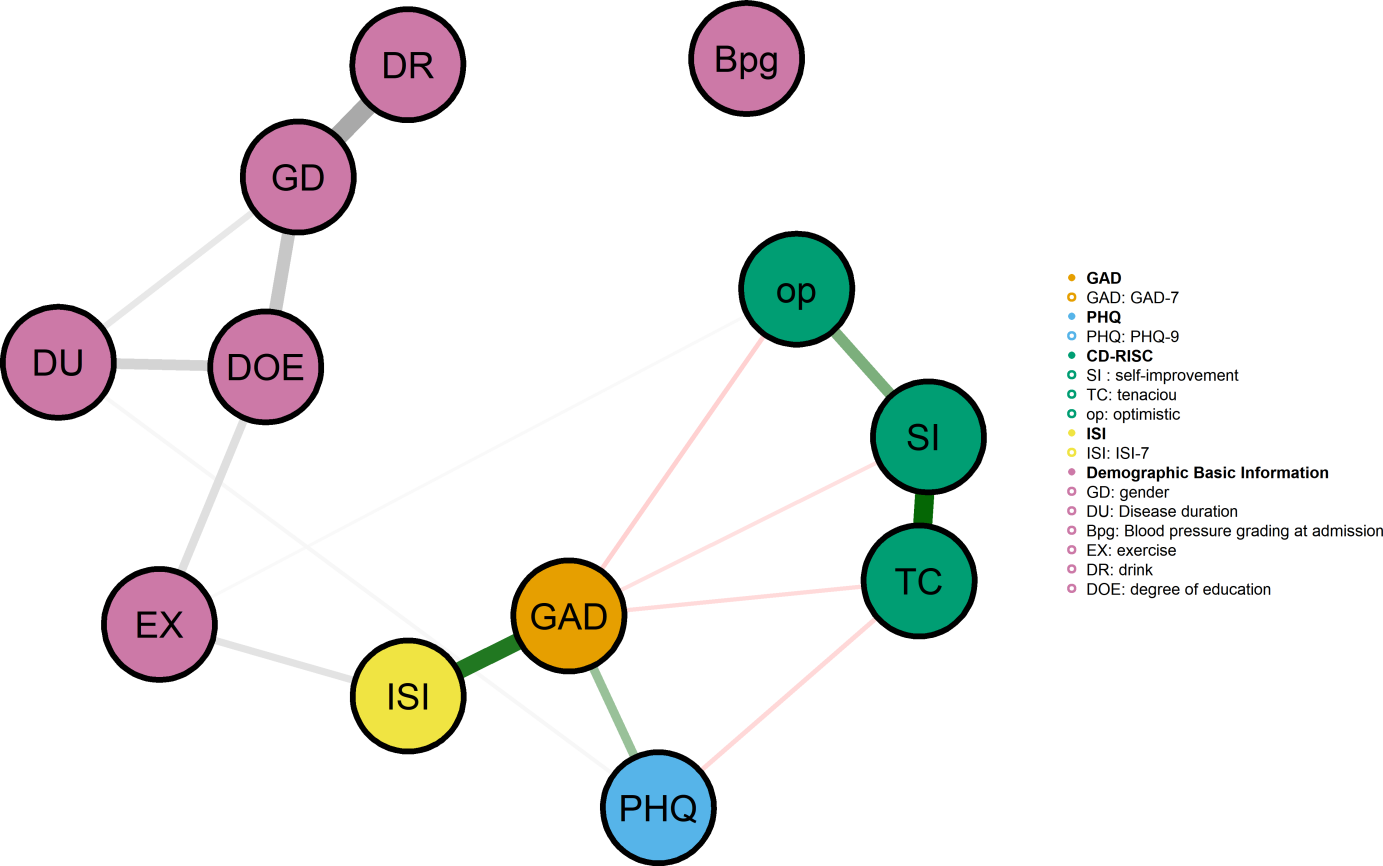
Comprehensive network model including depression, anxiety, and modifiable risk factors. Integrated network model depicting the associations among Patient Health Questionnaire-9 (PHQ-9), Generalized Anxiety Disorder Scale-7 (GAD-7), nsomnia Severity Index-7 (ISI-7), and modifiable psychological factors in hypertensive older adults (N = 562). Nodes represent: SI (Self-improvement), TC (Tenacious coping), op (Optimism), as well as demographic and clinical variables including GD (Gender), DU (Disease duration), Bpg (Blood pressure grading at admission), EX (Exercise), DR(Drink), and DOE(Degree of education). Node sizes are proportional to their strength centrality in the network. Edge thickness represents the magnitude of regularized partial correlations, with thicker edges indicating stronger conditional associations. Edge colors distinguish between positive associations (green) and negative associations (red).

The comprehensive network model incorporating Patient Health Questionnaire-9 (PHQ-9), Generalized Anxiety Disorder-7 (GAD-7), Insomnia Severity Index-7 (ISI-7), modifiable psychological factors, and demographic/clinical variables is presented in **Figure 5**. This expanded network comprised 12 nodes. Strong positive associations were observed between depression and anxiety symptoms (PHQ-GAD), and between insomnia and both depression (ISI-PHQ) and anxiety (ISI-GAD). There are edge connections between exercise (EX) and ISI, disease course (DU), and gender (GD). Drink (DR) is positively correlated with GD, while degree of education (DOE) is connected within demographic clusters and has an edge with GD, adjacent to the position of EX.

## 4 Discussion

This retrospective network analysis provides new insights into the symptom level structure of depression in elderly hypertensive individuals, identifying central symptoms and their interrelationships with anxiety, insomnia, and psychological resilience. Our research findings deepen our understanding of the heterogeneity of depression among this clinically vulnerable population and provide evidence-based targets for targeted interventions.

### 4.1 Anhedonia as the central node: mechanisms and clinical implications

Our network analysis identified anhedonia as the most central symptom, exhibiting the highest strength centrality. This finding extends prior network research in general older populations by demonstrating the prominent role of hedonic deficits specifically among older adults with hypertension. The salience of anhedonia in this population may reflect distinct neurobiological mechanisms linking cardiovascular pathophysiology to depression (22). A growing body of evidence suggests that hypertension-related cerebrovascular changes—including white matter hyperintensities and subcortical microvascular lesions—preferentially affect frontal-striatal reward circuitry, resulting in dopaminergic dysfunction and diminished hedonic capacity (22, 23). From a clinical perspective, the central positioning of anhedonia implies that successful amelioration of this symptom may yield broader network-wide improvements by deactivating interconnected symptoms. Concentration difficulties and fatigue emerged as additional core symptoms with high expected influence. The prominence of these cognitive and somatic symptoms reflects the multidimensional nature of depression in older adults with comorbid conditions. Fatigue, in particular, may represent a shared manifestation of both depressive and cardiovascular pathophysiology, establishing a bidirectional relationship that perpetuates symptom burden (24). The strong marginal weight (0.29) between fatigue and depression emphasizes this interconnection and suggests that interventions targeting energy levels may have downstream effects on emotional disorders. The strongest connection in our network is between sleep problems and anhedonia (weight=0.40), highlighting the crucial role of sleep disorders in the depressive symptoms of this population. Sleep disorders are common in both hypertension and depression, and may be a common pathway connecting these diseases (25). This finding suggests that sleep interventions, such as cognitive-behavioral therapy for insomnia (CBT-I) or sleep hygiene education, could be particularly effective in disrupting the depressive network (26). The study by Pyo et al. (27) identified “anhedonia” as the most central symptom in the Geriatric Depression Scale for Alzheimer’s disease patients, while “emptiness” was found to be the predominant symptom in patients with mild cognitive impairment. In this study, the depressive symptom network structure in elderly hypertensive patients differed from previous research on general elderly depression populations. In the elderly hypertensive patients in this study, anhedonia and fatigue exhibited more pronounced centrality. This difference may be related to the unique pathophysiological mechanisms of hypertensive patients: chronic vascular damage affects the function of the prefrontal-striatal reward circuit, thereby making anhedonia a more prominent core symptom. The findings suggest that depressive intervention strategies for elderly hypertensive patients may need to differ from those for the general elderly population, with a greater emphasis on targeted treatment of anhedonia and fatigue symptoms.

### 4.2 The depression-anxiety-insomnia triad: transdiagnostic considerations

The extended network model demonstrates a robust positive correlation linking depression, anxiety, and insomnia, which together constitute an interrelated triad of psychiatric symptoms. Bidirectional pathways emerging from our network analysis challenge conventional disease-specific boundaries, lending support to a transdiagnostic framework in which shared maintenance factors—cognitive rigidity, intolerance of uncertainty, and autonomic dysregulation—cut across traditional diagnostic categories (28). These findings carry notable therapeutic weight. Rather than adhering strictly to diagnosis-specific protocols, interventions that target overlapping mechanisms may yield broader clinical benefits. The Unified Protocol, a transdiagnostic cognitive-behavioral approach designed to address the common cognitive and behavioral underpinnings of emotional disorders, exemplifies this strategy and has demonstrated efficacy for co-occurring anxiety and depressive symptomatology (28). he depression-anxiety linkage (PHQ-GAD) identified here also informs treatment selection at both pharmacological and psychotherapeutic levels (29). From a psychopharmacological standpoint, selective serotonin reuptake inhibitors (SSRIs)—established first-line agents for depression and anxiety in older adults with favorable cardiovascular safety profiles—appear well suited given their dual-target efficacy (30). On the psychotherapy front, cognitive-behavioral interventions addressing both anxious and depressive cognitions may outperform protocols narrowly focused on a single disorder (31).

### 4.3 Psychological resilience: a modifiable protective factor

Correlation analyses revealed that each psychological resilience dimension—self-strengthening, hardiness, and optimism—was inversely associated with depressive symptom severity. Such findings suggest that resilience functions as a modifiable protective factor capable of buffering against depression among individuals living with chronic cardiovascular conditions (32). Conceptually, resilience reflects an individual’s capacity to adapt in the face of adversity, drawing on cognitive flexibility, effective coping repertoires, social connectedness, and a coherent sense of purpose. Several mechanistic pathways may underlie its protective effects. At the neurobiological level, resilient individuals tend to exhibit stronger prefrontal regulation of limbic reactivity, greater neuroplastic capacity, and more adaptive hypothalamic-pituitary-adrenal (HPA) axis responses to stressors (33). Behaviorally, resilience fosters problem-focused coping, sustained engagement in health-promoting activities, and preservation of social ties—patterns that counteract the withdrawal and inactivity central to depressive phenomenology. What distinguishes resilience from other depression risk factors commonly observed in older adults—such as multimorbidity or cognitive decline—is its amenability to intervention.

### 4.4 Suicidal ideation: peripheral position and clinical vigilance

Suicidal ideation emerged as the least central node in our network, occupying a relatively peripheral position within the symptom architecture. Although this finding in no way diminishes the clinical imperative to assess and manage suicidal thoughts, it does suggest that in this population suicidal ideation may arise downstream of other core symptoms rather than serving as a primary driver of network dynamics (34). This pattern carries implications for suicide prevention. Interventions that effectively target high-centrality symptoms—anhedonia, sleep disturbance, fatigue—may attenuate suicidal ideation indirectly by disrupting the symptomatic pathways through which it develops. That said, the peripheral network position of suicidal ideation must not translate into clinical complacency. Regardless of centrality metrics, any expression of suicidal thought warrants immediate evaluation and appropriate risk management. The network perspective simply implies that integrating suicide-focused assessment with interventions aimed at upstream, higher-centrality symptoms may represent the most effective preventive strategy (35).

### 4.5 Adjustable risk factors and the connection characteristics of symptom clusters

Network analysis revealed that exercise (EX) connected to the PHQ-GAD symptom cluster principally through insomnia severity (ISI), a pattern consistent with accumulating evidence on the mediating role of sleep in the exercise–depression relationship (36, 37). Barham et al. (37) showed that structured exercise interventions improve sleep quality, which in turn facilitates reductions in depressive symptoms. The network topology observed here provides a visual representation of this mechanistic pathway within an elderly hypertensive cohort., whereby exercise attenuates physiological hyperarousal and, consequently, anxiety and depressive symptoms. Future trials might therefore incorporate sleep quality as a mediating outcome to clarify the pathways linking exercise to mood improvement in this population. Regarding alcohol consumption, our findings suggest that isolated alcohol-reduction counseling is unlikely to yield direct antidepressant effects in elderly hypertensive patients. Reid et al. (38), which suggests that exercise reduces arousal levels, anxiety, and depressive symptoms to promote sleep. Future intervention studies may consider incorporating sleep quality as a mediating outcome indicator to better understand the mechanisms by which exercise affects depression in this population. Furthermore, from a clinical perspective, the findings of this study suggest that a standalone alcohol reduction intervention may not directly alleviate depressive symptoms in elderly hypertensive patients. Guertler et al. (39) observed a U-shaped pattern: both abstainers and heavier drinkers exhibited more severe depressive symptoms and elevated odds of major depressive disorder relative to moderate consumers. Additionally, it is important to note the impact of blood pressure levels on patients. Studies have indicated an association between circadian blood pressure patterns and clinical outcomes of EVT for acute ischemic stroke AIS (40). Postoperative nocturnal systolic blood pressure may provide additional information regarding the clinical prognosis of AIS patients after EVT. Integrated lifestyle interventions—simultaneously promoting physical activity, optimizing sleep, and moderating alcohol intake—may therefore offer greater therapeutic leverage than single-target approaches. Degree of education (DOE) occupied a peripheral network position, clustering with demographic variables yet lacking direct edges to symptom nodes. This topology accords with social-determinants-of-health frameworks, which conceptualize educational attainment as shaping health outcomes indirectly through pathways such as health literacy, resource availability, and behavioral patterns (41). Notably, DOE’s proximity to EX in the network raises the possibility that education influences depression largely via its impact on physical activity engagement. Higher educational attainment is associated with more regular exercise, healthier lifestyle adoption, and better healthcare access. These observations underscore the need for public health strategies that deliver tailored health education and behavioral support to less-educated older adults, thereby ensuring equitable access to the protective benefits of exercise and sleep-focused programs.

### 4.6 Strengths, limitations, and future directions

This study possesses several methodological strengths. We employed psychometrically validated instruments, applied rigorous network estimation with systematic stability checks, and examined clinically meaningful moderating variables. Recruitment from tertiary general hospitals enhances the generalizability of our findings within the Chinese healthcare context. Several limitations nonetheless merit consideration. The cross-sectional, retrospective design precludes causal inference regarding temporal dynamics among symptoms. Although network theory posits directional relationships, our data capture only contemporaneous association patterns from a single assessment window. Moreover, the retrospective nature of data extraction introduces potential selection bias, as analyses were restricted to patients with complete evaluations on record. Future research should employ prospective longitudinal designs—ideally incorporating ecological momentary assessment (EMA) or repeated-measures protocols spanning multiple time points and seasons—to test the robustness of the present findings. Such designs would permit examination of symptom activation cascades and identification of sentinel symptoms that predict subsequent clinical deterioration.

## 5 Conclusions

This network analysis reveals the complex, interconnected architecture of depressive symptoms in older adults with hypertension, identifying anhedonia, concentration difficulties, and fatigue as central symptoms within the network. The close interrelationships observed among depressive, anxiety, and insomnia symptoms, coupled with the protective role of psychological resilience, underscore the need for comprehensive, transdiagnostic approaches to mental health care in this population. From a clinical standpoint, these findings offer practical guidance for depression screening and intervention in primary care and community settings. Community health service centers may consider adopting streamlined screening instruments that prioritize the assessment of core symptoms, thereby facilitating efficient case identification.

## Data Availability

The original contributions presented in the study are included in the article/supplementary material. Further inquiries can be directed to the corresponding authors.
